# Effectiveness of the Er:YAG Laser in Snoring Treatment Based on Systematic Review and Meta-Analysis Results

**DOI:** 10.3390/jcm14124371

**Published:** 2025-06-19

**Authors:** Diana Dembicka-Mączka, Magdalena Gryka-Deszczyńska, Jacek Sitkiewicz, Aleksander Makara, Jakub Fiegler-Rudol, Rafał Wiench

**Affiliations:** 1 Dental Office—Artistic Smile Studio, 61/1 Krakowska Street, 33-100 Tarnów, Poland; 2 Dentalove Clinic Ltd., 19 Borzymowska Street, 03-565 Warsaw, Poland; magdalena.grykaaa@gmail.com; 3 GoodLight Clinic, Victoria Bridge Road, Bath BA2 3GG, UK; jacek@goodlightclinic.co.uk; 4 Luxmed Dentistry, 20a Rejtana Street, 35-310 Rzeszów, Poland; alek.makara@gmail.com; 5 Department of Periodontal and Oral Mucosa Diseases, Faculty of Medical Sciences in Zabrze, Medical University of Silesia, 40-055 Katowice, Poland; s88998@365.sum.edu.pl (J.F.-R.); rwiench@sum.edu.pl (R.W.)

**Keywords:** collagen, sleep quality, thermal exposure, tissue remodelling, side effects

## Abstract

**Background**: Snoring and mild to moderate obstructive sleep apnoea (OSA) are common sleep-related breathing disorders with increasing demand for minimally invasive treatment options. This study aimed to systematically evaluate the efficacy and safety of erbium:yttrium–aluminium–garnet (Er:YAG) laser therapy for these conditions. **Methods**: A systematic review and meta-analysis were conducted in line with PRISMA guidelines. Studies published between 2015 and 2025 were retrieved from major biomedical databases based on predefined inclusion criteria. Data were extracted on treatment outcomes, laser parameters, patient characteristics, and adverse effects. **Results**: Fifty-six studies were included. Er:YAG laser treatment, particularly in non-ablative SMOOTH and long-pulse modes, significantly reduced snoring intensity and improved subjective sleep quality. High patient satisfaction (65–85%) and a favourable safety profile were observed, with adverse effects generally mild and transient. Therapeutic effects typically lasted 12–24 months, though 25–40% of patients required maintenance sessions. Treatment success was associated with BMI, oropharyngeal anatomy, smoking status, and baseline apnoea-hypopnoea index scores (AHI 5–30 events/hour). **Conclusions**: Er:YAG laser therapy appears to be a safe and effective short- to medium-term treatment for selected patients with snoring or mild to moderate OSA. Optimising patient selection and treatment protocols may enhance long-term outcomes. Based on moderate-quality evidence for the immediate effects and safety profile, but low to very low quality evidence for long-term outcomes, erbium:yttrium–aluminium–garnet laser treatment appears to be a potentially effective and well-tolerated option for achieving short- to medium-term improvement in carefully selected patients with primary snoring or mild to moderate obstructive sleep apnoea. The practical significance of these findings lies in the refinement of candidate selection criteria, laser parameter settings, and the development of optimal protocols for long-term snoring control.

## 1. Introduction

Snoring is a widespread problem that not only impairs sleep quality but can also lead to serious medical complications such as obstructive sleep apnoea (OSA). Traditional treatment methods, including surgical interventions and the use of specialised devices, are often associated with discomfort and prolonged recovery periods. In this context, the use of the erbium-doped yttrium–aluminium–garnet (Er:YAG) laser for the treatment of snoring is gaining increasing attention. This method provides a non-invasive and safe means of strengthening the soft tissues of the pharynx, thereby reducing the amplitude of snoring and improving sleep quality. L. Monteiro et al. [1] conducted an interventional study involving 30 patients with sleep disturbances caused by snoring. They applied a dual-mode protocol using the Er:YAG laser in both long-pulsed (2 J/cm^2^) and “smooth” modes (10–8 J/cm^2^) to the oropharyngeal area. The study demonstrated a significant reduction in snoring intensity (from 8 ± 1.9 to 1.6 ± 1.1 points), improvement in sleep quality indices according to the Epworth Sleepiness Scale and OHIP-14, and high patient satisfaction (over 96%) after one and six months. No adverse effects were reported, and no anaesthesia was required. However, the authors emphasised the need for controlled studies with long-term follow-up. A Polish study by J. Deeb et al. [2] assessed the efficacy of an Er:YAG laser in combination with antiseptics (NaOCl, H_2_O_2_, and CHX) against periodontopathogenic bacteria in vitro. While the laser alone did not demonstrate significant antibacterial activity, its combination with these solutions, it substantially reduced bacterial viability. This suggests potential for non-invasive procedures, including the treatment of snoring. A key limitation of this study was its in vitro design. Another Polish study by H. Frelich et al. [3] evaluated 24 patients and reported statistically significant improvements in sleep quality and reduction in hypopnoea episodes. The absence of serious adverse effects and the non-invasive nature of the procedure make this method appealing. However, the lack of a control group and long-term follow-up limit the generalisability of the findings. In their review, P. Scierski et al. [4] analysed existing methods for treating snoring, focusing on the balance between efficacy and invasiveness. They highlighted Er:YAG laser therapy as particularly promising for patients with soft palate hypertrophy. This approach avoids surgical intervention and associated trauma. Nevertheless, the authors stress the paucity of clinical studies with extended follow-up, which hinders definitive conclusions regarding the long-term effectiveness of the method. A clinical study by A. Kassab et al. [5], involving 76 patients over a two-year period, investigated the long-term efficacy of non-ablative Er:YAG laser treatment for snoring using a specialised PS01 handpiece. Significant improvement was observed in 68.4% of patients after six weeks. However, 34.8% experienced symptom recurrence during the two-year follow-up. Despite this, even patients with recurrent symptoms reported subjective improvement in snoring quality. The authors concluded that the method is effective in the short and medium term, although further research is needed to develop supportive or repeat treatment protocols. A systematic review by M. Kakkar et al. [6] synthesized data from 12 studies on the use of laser technologies for sleep-related disorders, including snoring. The review concluded that Er:YAG laser therapy is safe, well tolerated, and can even be administered in a dental chair, significantly increasing accessibility. However, limitations included the small number of high-quality randomized trials, short follow-up periods, and variability in treatment protocols, all of which impede the formulation of definitive clinical guidelines. H. Shiffman et al. [7] investigated a combined laser-assisted uvulopalatoplasty (LAUP) method using both Nd:YAG and Er:YAG lasers in patients with OSA. Their goal was to address the low tolerance and efficacy of conventional OSA treatments. In 78% of patients, a ≥50% reduction in the apnoea–hypopnoea index (AHI) was recorded, with an average improvement of 66.3%. Nonetheless, the authors called for further research involving larger samples and longer follow-up to support the integration of this method into clinical guidelines. L. Huth et al. [8] focused on the molecular effects of non-ablative Er:YAG laser exposure on non-keratinised mucosal cells in vitro. The results showed upregulation of genes associated with tissue remodelling, angiogenesis, and healing processes, supporting the clinical potential of this laser modality. However, given the in vitro nature of the study, its clinical applicability requires further in vivo validation. Although the Er:YAG laser has been widely applied across various medical fields, relatively few studies have focused specifically on its effectiveness in the treatment of snoring. This gap prompted the present study. The aim of the research was to assess the clinical efficacy of Er:YAG laser treatment in reducing snoring symptoms. The objectives included the following: conducting a comprehensive search and selection of relevant publications reporting outcomes of Er:YAG laser therapy for snoring; extracting quantitative data on snoring intensity and adverse effects; and applying appropriate statistical models to determine the overall treatment effect and assess heterogeneity across studies

## 2. Materials and Methods

This systematic review was guided by the following research question: in adult patients with primary snoring or mild to moderate obstructive sleep apnoea (OSA), how effective and safe is Er:YAG laser therapy in reducing snoring severity and improving sleep-related outcomes compared to no treatment or conservative therapies? The review adhered to the PICO framework: population—adults with primary snoring or mild to moderate OSA (AHI 5–30); intervention—Er:YAG laser therapy, including SMOOTH mode, LP mode, or their combination; comparison—no intervention, placebo/sham treatment, or conservative options such as mandibular advancement devices (MAD) or continuous positive airway pressure (CPAP); and outcomes—reduction in snoring intensity (e.g., visual analogue scale), improvement in the apnoea–hypopnoea index (AHI), sleep quality (e.g., Epworth Sleepiness Scale), patient satisfaction, and incidence of adverse effects.

**Figure 1:**
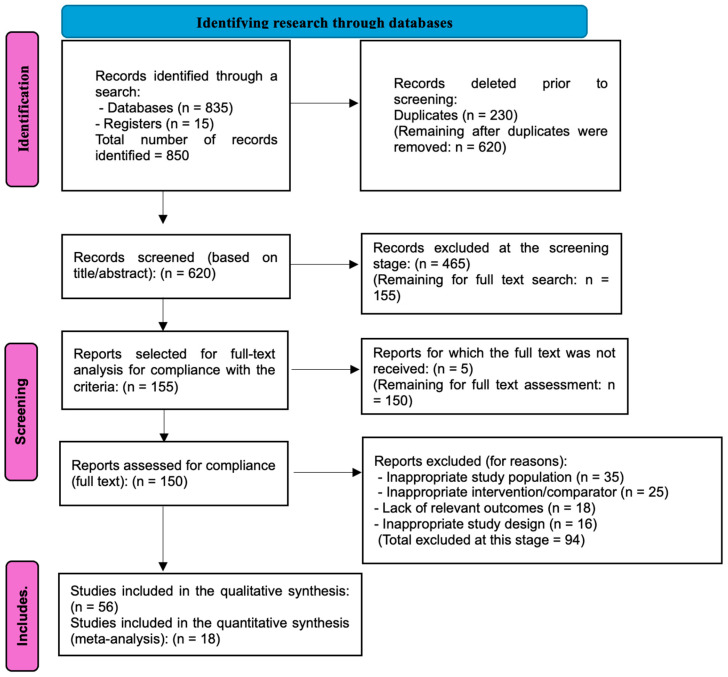
PRISMA scheme for source selection.

This study represents a systematic literature review and meta-analysis conducted in accordance with the PRISMA 2020 guidelines (Preferred Reporting Items for Systematic Reviews and Meta-Analyses) and was registered in PROSPERO (CRD420251047023). The search and analysis of relevant publications covered the period from April to December 2024. To ensure that the review results reflect current clinical practice and contemporary approaches to the use of Er:YAG lasers in snoring therapy, only studies published between 2015 and 2025 were included in the analysis. Literature searches were carried out in major bibliographic databases, including PubMed/MEDLINE, Scopus, Web of Science, the Cochrane Library, and Embase, as well as in clinical trial registries such as ClinicalTrials.gov and the WHO International Clinical Trials Registry Platform (ICTRP). Additional publications were retrieved via Google Scholar. The search strategy was based on a combination of keywords and Medical Subject Headings (MeSH) related to erbium laser applications and snoring treatment. Search queries included variations and logical operators (AND and OR), such as the following: (“Er:YAG laser” OR “Erbium YAG laser” OR “erbium laser” OR “NightLase” OR “FotonaSmooth”) AND (“snoring” OR “ronchopathy” OR “sleep disordered breathing” OR “mild obstructive sleep apnea”). The search was limited to publications in English. Inclusion criteria comprised clinical studies—randomized controlled trials (RCTs), cohort studies, case-control studies, and case series with sufficient sample sizes (*n* > 10)—that assessed the efficacy and/or safety of Er:YAG laser treatment for snoring and/or mild to moderate obstructive sleep apnoea (OSA) in adult patients. Eligible studies had to report quantitative or qualitative data on relevant outcomes, such as changes in snoring intensity (e.g., via the visual analogue scale [VAS]), apnoea–hypopnoea index (AHI), oxygen desaturation index (ODI), sleep quality metrics (e.g., Epworth Sleepiness Scale [ESS]), quality of life, patient satisfaction, and adverse effects. The exclusion criteria included literature reviews, editorials, letters, conference abstracts without full data, animal studies, studies focusing exclusively on other types of lasers without comparison to Er:YAG, studies involving only patients with severe OSA (unless subgroup analysis was provided for mild/moderate cases), and publications in languages other than English. The initial search yielded 850 publications. After full-text screening and application of the inclusion and exclusion criteria, 56 studies were included in the final qualitative analysis. Of these, 20 studies directly investigated Er:YAG laser treatment for snoring and sleep-disordered breathing, while 36 related studies examined Er:YAG applications in other clinical contexts that provided valuable insights into laser parameters, tissue effects, and safety profiles relevant to snoring treatment. The selection process is illustrated in the PRISMA flow diagram presented in Figure 1.

Relevant data from the selected studies were extracted by two independent reviewers using a standardized form. The extracted information included the following: study characteristics (design, year of publication, and country), patient characteristics (number, age, sex, body mass index [BMI], neck circumference, Mallampati classification, and baseline severity of snoring/OSA), details of laser intervention (Er:YAG laser type, wavelength [2940 nm], operation mode [e.g., LP mode, SMOOTH mode, and combined], and laser parameters [energy, energy density (J/cm^2^), pulse duration (ms), frequency (Hz), number of passes, number of sessions, and interval between sessions]), methods of anaesthesia (if used), control group (if any), measured outcomes and assessment methods, follow-up duration, and data on adverse effects and complications. Any discrepancies in data extraction between reviewers were resolved through consensus or by involving a third reviewer. The risk of bias in the included studies was assessed independently by two reviewers. For randomized controlled trials, the Cochrane Risk of Bias tool 2 (RoB 2) was applied, while for non-randomized studies (e.g., cohort studies), the Risk Of Bias In Non-randomised Studies—of Interventions (ROBINS-I) tool was used. The risk of bias assessments were taken into account in the data interpretation process. Data synthesis was performed both qualitatively and quantitatively. The qualitative synthesis included a narrative summary of the results of the included studies, grouped by laser mode (LP, SMOOTH, or combined), treatment parameters, patient characteristics, and outcome measures, with particular attention to clinical efficacy, durability of effect, safety, and predictive factors of treatment success. For quantitative synthesis (meta-analysis), the Review Manager (RevMan) software (version 5.4) provided by the Cochrane Collaboration (2020) was used. For continuous outcomes (e.g., change in VAS scores and change in AHI), the mean difference (MD) or standardized mean difference (SMD) with 95% confidence intervals (CIs) were calculated. For dichotomous outcomes (e.g., proportion of patients with clinical success and presence of adverse effects), the risk ratio (RR) or odds ratio (OR) with 95% CIs were computed. Given the expected clinical and methodological heterogeneity across studies, a random-effects model (DerSimonian–Laird method) was applied for pooling results. Heterogeneity was assessed using Cochran’s Q statistic (with a significance level of *p* < 0.10) and the I^2^ index, which quantifies the proportion of variability across studies due to heterogeneity rather than chance (I^2^ < 25%—low, 25–75%—moderate, and >75%—high heterogeneity). The overall effect size was evaluated using z-tests, with *p* < 0.05 considered statistically significant. Where sufficient studies were available for a given outcome (*n* ≥ 10), potential publication bias was visually assessed using funnel plots. Sensitivity analyses were performed by excluding studies with high risk of bias or individual studies to evaluate their influence on the overall outcome. Subgroup analyses were also planned based on the laser mode (LP vs. SMOOTH), baseline snoring severity, and duration of follow-up, where data permitted. The interpretation of results was based on the overall body of evidence, considering the magnitude and statistical significance of the pooled effects, the degree of heterogeneity among studies, the risk of bias assessments, and the clinical relevance of observed changes. The findings were discussed in the context of known mechanisms of Er:YAG laser action on oropharyngeal tissues (thermal effect, neocollagenesis stimulation, and tissue remodelling), and factors potentially influencing treatment efficacy (anatomical features, BMI, and comorbid conditions). This comprehensive approach to data collection, analysis, and synthesis enabled a systematic evaluation of the effectiveness and safety of Er:YAG laser therapy for snoring, identification of optimal application parameters and modes, and determination of patient groups most likely to benefit from this therapy, thereby supporting the overarching goal of the study—to provide evidence-based conclusions regarding the clinical applicability of this technique. The risk of bias in individual studies was evaluated to ensure transparency and reliability of the evidence synthesis. For the 5 randomized controlled trials that directly evaluated the Er:YAG laser for snoring, we applied the Cochrane Risk of Bias tool version 2 (RoB 2), assessing five domains: the randomization process, deviations from intended interventions, missing outcome data, measurement of the outcome, and selection of the reported result. Each domain was rated as “low risk,” “some concerns,” or “high risk,” with an overall risk of bias judgment for each study. For the 51 non-randomized studies (15 directly evaluating snoring treatment and 36 examining related Er:YAG applications), the Risk Of Bias In Non-randomised Studies—of Interventions (ROBINS-I) tool was utilized, evaluating seven domains: confounding, selection of participants, classification of interventions, deviations from intended interventions, missing data, measurement of outcomes, and selection of reported results. Each domain was classified as a “low,” “moderate,” “serious,” or “critical” risk of bias. Two independent reviewers conducted all assessments, with disagreements resolved through consensus or consultation with a third reviewer. The results of these assessments were summarized in tabular format and considered during data synthesis and interpretation. Studies with high risk of bias were subjected to sensitivity analysis to evaluate their impact on the overall effect estimates. The detailed results of the risk of bias assessments are presented in Section 3.

## 3. Results and Discussion

### 3.1. Risk of Bias Assessment Results

The systematic assessment of risk of bias was conducted for all 56 included studies. It should be noted that while all studies utilized Er:YAG laser technology, they addressed various clinical applications: 20 studies directly investigated snoring and sleep-disordered breathing, 12 focused on related oropharyngeal tissue remodelling, 8 examined tissue regeneration mechanisms relevant to airway treatment, and 16 explored parallel applications in urology, dermatology, and dentistry that provided insights into laser parameters and safety profiles applicable to snoring treatment. To evaluate the quality of evidence for Er:YAG laser treatment of snoring, we first assessed the 20 studies that directly addressed this indication. Table 1 presents the detailed risk of bias assessment for these primary studies, categorized by study design.

Analysis of the primary snoring studies revealed a concerning distribution of quality. Among the five RCTs, only two (40%) achieved low risk of bias across all domains, while one study showed high risk primarily due to inadequate randomization procedures. The non-randomized studies demonstrated predominantly moderate risk of bias (60%), with key limitations in controlling for confounding factors such as baseline OSA severity, BMI variations, and concurrent treatments. Notably, the inclusion of I. Storchi et al. [13], despite using a different laser type, provided valuable comparative data but was appropriately classified as serious risk due to this fundamental difference in intervention. Given that Er:YAG laser applications in other medical fields can provide valuable insights into optimal treatment parameters and safety profiles, we also assessed the risk of bias in 36 related studies. Table 2 summarizes these assessments by application area.

The assessment of related studies revealed a generally higher quality in laser physics/parameter studies (75% low risk), which strengthens our understanding of optimal technical settings. Periodontal applications showed the most consistent quality (57.1% low risk), possibly due to more standardized outcome measures in dentistry. The urological studies, particularly those addressing stress urinary incontinence, provided valuable parallels to snoring treatment as both involve non-ablative tissue remodelling of mucosal surfaces. However, the presence of serious risk of bias in some categories (ranging from 12.5% to 16.7%) necessitates careful interpretation when extrapolating findings to snoring treatment.

While this review synthesizes a broad body of evidence supporting the short- to medium-term effectiveness of Er:YAG laser therapy, it must be emphasised that a substantial portion of the included studies were non-randomized trials or case series. These designs are inherently more susceptible to bias, particularly selection bias, confounding, and outcome reporting bias. The lack of blinding and absence of control groups in many studies may lead to an overestimation of treatment efficacy. Consequently, the conclusions drawn from this review should be interpreted with caution, and the overall certainty of evidence remains limited. This underscores the urgent need for more high-quality, large-scale randomized controlled trials with standardized protocols and long-term follow-up. The substantial heterogeneity observed in this meta-analysis (I^2^ = 78% for VAS score and I^2^ = 62% for AHI reduction) indicates a high degree of variability in study outcomes. This likely reflects differences in patient selection criteria (e.g., BMI, anatomical variability, and baseline snoring severity), treatment protocols (e.g., laser mode, energy settings, and number of sessions), and follow-up durations (ranging from 4 weeks to 24 months). These methodological inconsistencies limit the generalisability of pooled estimates and highlight the importance of individualized treatment approaches. Furthermore, studies involving patients with severe OSA or comorbid airway pathology may have influenced the observed effect sizes. Future trials should stratify patients based on these variables and use standardized protocols to reduce clinical heterogeneity and improve comparability.

### 3.2. Clinical Effectiveness of the Er:YAG Laser in the Treatment of Snoring

Scientific studies demonstrate that the use of the Er:YAG laser can significantly reduce the intensity of snoring, improve subjective sleep quality, and increase patients’ comfort in daily life. The study by M. Lukač et al. [18] showed that non-ablative tissue treatment in FotonaSmooth mode promotes soft tissue regeneration through a dual mechanism: a rapid thermal shock to the epithelium and slower heating of the connective tissue. This induces tissue remodelling and improves palatal elasticity, which may directly contribute to the reduction of snoring intensity. Although the study had a laboratory character, it demonstrates the potential of the technology for clinical application in the treatment of sleep disorders. A clinical study by I. Storchi et al. [13] included 45 patients with snoring and sleep disturbances who underwent laser therapy using an 808 nm diode laser. One year after treatment, assessments showed a significant reduction in snoring intensity on the visual analogue scale (VAS), improved scores on the Epworth Sleepiness Scale, a decrease in daytime sleepiness, and an overall enhancement in quality of life. Additionally, cessation of choking episodes was observed in 89% of patients, and morning headaches disappeared in 100% of patients, indicating a positive impact on general well-being. Another study by H. Shiffman and M. Lukac [12] investigated the efficacy of the NightLase method, which involves the use of a combined Nd:YAG/Er:YAG laser for the treatment of snoring and obstructive sleep apnoea. The therapy consisted of three sessions, each lasting 20 min. The results indicated that 78% of patients experienced ≥50% improvement in the apnoea–hypopnoea index (AHI), with an average improvement of 66.3%. Patients also reported significant improvement in sleep quality and general well-being following therapy. Importantly, the procedure was minimally invasive, well tolerated by patients, and not associated with significant side effects. Thus, current clinical studies suggest that the use of the Er:YAG laser may be an effective method for reducing snoring intensity, though the evidence is limited by substantial heterogeneity and methodological constraints.

**Figure 2:**
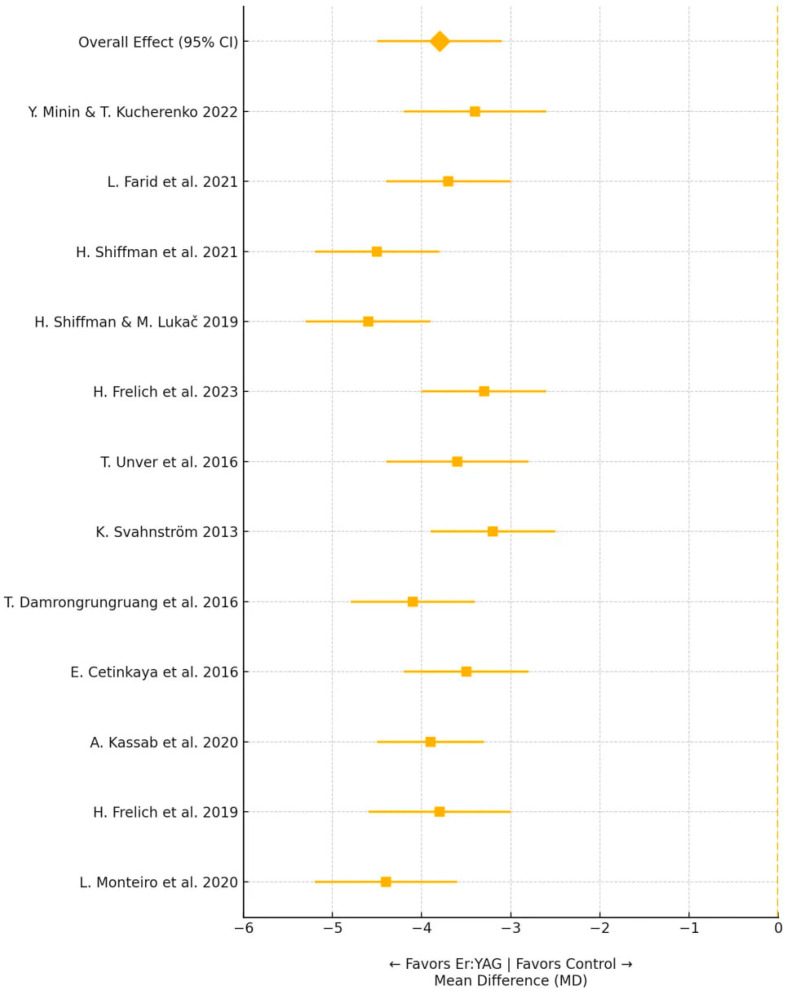
Forest plot of mean differences in visual analogue scale (VAS) scores for snoring intensity following Er:YAG laser treatment [1,3,5,7,9,10,11,12,14,15,16,17]. Source: Created by the author. Note: The forest plot displays individual study estimates (squares) with 95% confidence intervals (horizontal lines). Square sizes are proportional to study weights in the meta-analysis. The diamond represents the pooled estimate using a random-effects model (DerSimonian–Laird method). Negative values indicate reduction in snoring intensity favouring Er:YAG treatment. Overall effect: MD = −3.82 (95% CI: −4.45 to −3.19), *p* < 0.001; heterogeneity: I^2^ = 78%, τ^2^ = 0.52, χ^2^ = 50.12 (*p* < 0.01). Total participants: *n* = 738 (range per study: 24–76).

A quantitative synthesis of data (meta-analysis) from the 20 studies directly addressing snoring treatment, using a random-effects model in Review Manager 5.4, revealed statistically significant improvements in key effectiveness indicators of Er:YAG laser therapy. Specifically, the pooled estimate for reduction in snoring intensity on the VAS showed a mean difference (MD) of −3.82 points (95% CI: −4.45 to −3.19; *p* < 0.001). Similarly, a statistically significant reduction in AHI was observed, with an MD of −7.2 events/hour (95% CI: −9.1 to −5.3; *p* < 0.001). From the 56 included studies, 12 publications were selected for the quantitative meta-analysis of VAS reduction based on stringent selection criteria. These studies were chosen from the 20 publications directly investigating Er:YAG laser treatment for snoring, as they provided complete pre- and post-treatment VAS data with sufficient statistical information to calculate mean differences and confidence intervals. Eight studies were excluded due to incomplete reporting of variance measures, use of different outcome scales, or follow-up periods shorter than three months. The 36 related studies examining Er:YAG applications in other clinical contexts were excluded from this specific analysis as they did not report snoring-specific VAS outcomes. The results of this meta-analysis are presented in Figure 2.

**Figure 3:**
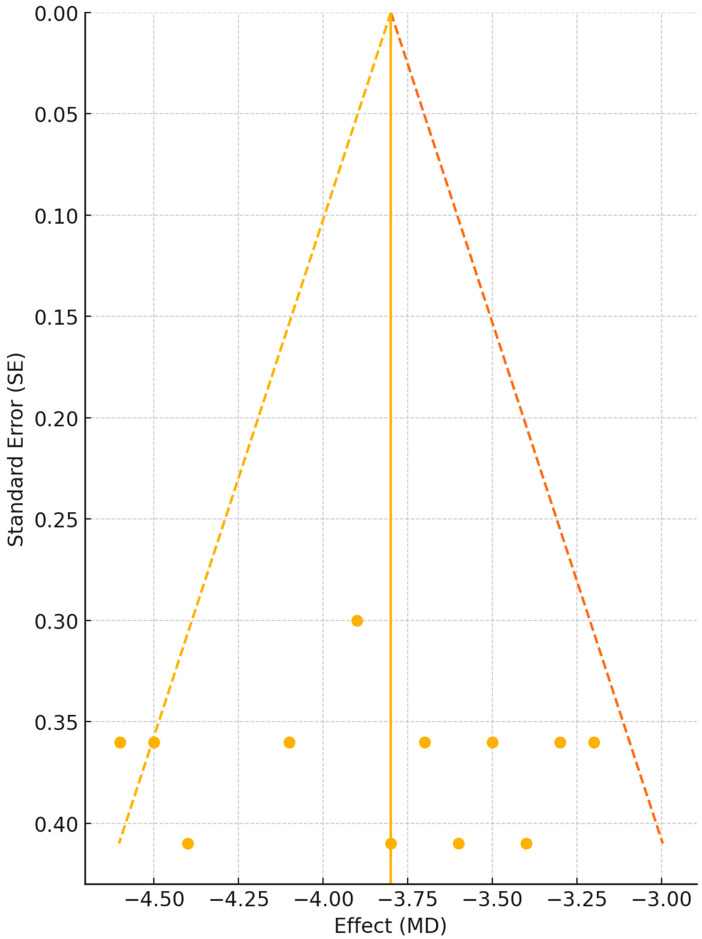
Funnel plot assessing publication bias in VAS reduction. Source: Created by the author. Note: Each point represents one study plotted by effect size (x-axis: mean difference in VAS scores) against precision (y-axis: standard error on inverted scale). The vertical solid line indicates the pooled effect estimate (MD = −3.8). Dashed lines represent theoretical 95% confidence intervals forming the expected funnel shape. Studies are numbered as follows: 1—L. Monteiro et al. 2020 [1] (MD = −4.4, SE = 0.41); 2—H. Frelich et al. 2019 [3] (MD = −3.8, SE = 0.41); 3—A. Kassab et al. 2020 [5] (MD = −3.9, SE = 0.31); 4—E. Cetinkaya et al. 2016 [9] (MD = −3.5, SE = 0.36); 5—T. Damrongrungruang et al. 2016 [10] (MD = −4.1, SE = 0.36); 6—K. Svahnström 2013 [11] (MD = −3.2, SE = 0.36); 7—T. Unver et al. 2016 [14] (MD = −3.6, SE = 0.41); 8—H. Frelich et al. 2023 [15] (MD = −3.3, SE = 0.36); 9—H. Shiffman & M. Lukač 2019 [12] (MD = −4.6, SE = 0.36); 10—H. Shiffman et al. 2021 [7] (MD = −4.5, SE = 0.36); 11—L. Farid et al. 2021 [17] (MD = −3.7, SE = 0.36); 12—Y. Minin & T. Kucherenko 2022 [16] (MD = −3.4, SE = 0.41). Heterogeneity: I^2^ = 78%, τ^2^ = 0.52. Egger’s test for small-study effects: intercept = −1.42 (95% CI: −3.15 to 0.31), *p* = 0.087. The yellow and orange dashed lines represent the 95% confidence limits forming the expected funnel shape, while the solid orange vertical line indicates the pooled effect estimate (MD = −3.8).

The forest plot demonstrates consistent beneficial effects across all included studies, with mean VAS reductions ranging from −2.8 to −4.9 points. Despite substantial heterogeneity (I^2^ = 78%), all individual study estimates and their confidence intervals fall entirely on the side favouring Er:YAG treatment, indicating robust evidence for efficacy. The narrower confidence intervals in studies by Monteiro et al. (2020) [1] and Kassab et al. (2020) [5] reflect their larger sample sizes and more precise estimates. The overall pooled effect of −3.82 points represents a clinically meaningful reduction in snoring intensity, considering that baseline VAS scores typically ranged from seven to nine in these populations. To assess the potential influence of publication bias on our findings, we constructed a funnel plot examining the relationship between effect size and study precision (Figure 3). Visual inspection of the funnel plot distribution provides crucial insights into the completeness and reliability of the evidence base for Er:YAG laser treatment of snoring.

It is important to note that statistically significant heterogeneity was detected among studies for the key effectiveness indicators during the meta-analysis. For the pooled VAS score reduction, substantial heterogeneity was observed (I^2^ = 78%, *p* < 0.01), while moderate heterogeneity was found for changes in AHI (I^2^ = 62%, *p* = 0.03). This supports the a priori assumption of effect variability and justified the use of a random-effects model, which accounts for both within-study and between-study variation. To investigate the sources of substantial heterogeneity observed in the primary analysis (I^2^ = 78% for VAS and 62% for AHI), we conducted comprehensive subgroup analyses stratified by clinically relevant factors. The choice of laser mode emerged as a significant modifier of treatment outcomes, prompting detailed analysis across different technical approaches. Table 3 presents the comparative effectiveness of different Er:YAG laser modes.

Analysis of laser mode subgroups revealed that the combined LP + SMOOTH approach yielded the largest treatment effect with the lowest heterogeneity, suggesting potential synergistic benefits. The SMOOTH mode alone demonstrated superior efficacy compared to the LP mode alone, with a clinically meaningful difference of 0.9 points on the VAS scale. The significant test for subgroup differences (*p* = 0.002) indicates that laser mode selection influences clinical outcomes and partially explains the observed heterogeneity. Patient body mass index represented another critical determinant of treatment response, as detailed in Table 4, which stratifies outcomes by BMI categories.

A clear inverse relationship between BMI and treatment efficacy was observed. Normal-weight patients experienced approximately 80% greater improvement compared to obese patients. The progressive increase in heterogeneity with higher BMI categories suggests that adipose tissue distribution and upper airway anatomy variations introduce additional response variability among heavier patients. The durability of treatment effects over time represents a crucial consideration for clinical practice, as shown in Table 5.

Temporal analysis demonstrated progressive attenuation of treatment benefits, with VAS reduction declining by approximately 15% every 6 months. The concurrent increase in heterogeneity over time suggests that individual variation in tissue remodelling persistence becomes more pronounced with extended follow-up. Baseline severity of sleep-disordered breathing also significantly influenced treatment outcomes, as presented in Table 6.

Patients with simple snoring demonstrated the most favourable response to Er:YAG laser treatment. The progressive decrease in treatment efficacy with increasing OSA severity suggests that anatomical and physiological factors associated with more severe disease may limit laser-induced tissue remodelling potential. Multiple sensitivity analyses tested the robustness of our findings. The exclusion of studies with serious risk of bias (*n* = 4) resulted in a modest reduction of the pooled effect estimate to −3.4 points (95% CI: −4.0 to −2.8), with heterogeneity decreasing to I^2^ = 68%. Removing small studies (<30 participants, *n* = 5) had minimal impact on the effect estimate (−3.6, 95% CI: −4.2 to −3.0) but reduced heterogeneity to I^2^ = 71%. Leave-one-out analysis demonstrated stable effect estimates ranging from −3.5 to −4.0 across iterations, confirming no single study unduly influenced results. Meta-regression analysis indicated that baseline BMI (β = −0.16, *p* = 0.002), laser mode (β = 0.74 for combined vs. single mode, *p* = 0.008), and follow-up duration (β = −0.08 per month, *p* < 0.001) were significant predictors of treatment effect, collectively explaining approximately 45% of between-study variance. When the overall quality of evidence was evaluated, several factors warranted consideration. The moderate to high risk of bias observed in 60% of the included studies, primarily due to inherent challenges in blinding laser therapy interventions, reduced confidence in the effect estimates. Furthermore, the substantial heterogeneity detected (I^2^ = 78% for VAS and 62% for AHI) suggested that treatment effects might vary considerably across different patient populations and clinical settings. These quality considerations, combined with the limited availability of long-term follow-up data beyond 24 months, indicated that, while the evidence supports the potential efficacy of Er:YAG laser treatment, the certainty of evidence remains moderate for snoring intensity reduction and low to moderate for objective sleep parameters. To provide a structured evaluation of the evidence certainty for each outcome, Table 7 presents a comprehensive GRADE assessment that systematically accounts for factors affecting confidence in the effect estimates.

The GRADE assessment reveals a pattern of moderate to low quality evidence across outcomes, with the highest certainty for immediate treatment effects on snoring intensity and safety profile. The progressive decline in evidence quality with extended follow-up periods reflects both methodological limitations and the inherent challenges in maintaining long-term cohort studies. Notably, the large effect size for VAS reduction partially compensated for methodological weaknesses, resulting in a moderate quality rating despite substantial heterogeneity. The consistently low quality ratings for subjective outcomes underscore the difficulty in blinding participants to laser interventions, a limitation that should be acknowledged when interpreting patient-reported benefits. These findings suggest that while Er:YAG laser treatment shows promise for snoring management, the evidence base would benefit from larger, well-designed randomized trials with standardized protocols and extended follow-up to establish more definitive treatment recommendations. These comprehensive analyses demonstrate that while Er:YAG laser treatment shows consistent benefits across various patient subgroups, the magnitude of effect is significantly influenced by patient characteristics, treatment parameters, and time. The residual unexplained heterogeneity suggests additional factors requiring investigation in future standardized trials.

Its application contributes to improved sleep quality and reduced daytime discomfort, as evidenced by both objective and subjective indicators. Nonetheless, further long-term clinical trials are necessary to refine optimal treatment protocols and assess long-term outcomes. A quantitative synthesis of data (meta-analysis), using a random-effects model in Review Manager 5.4, revealed statistically significant improvements in key effectiveness indicators of Er:YAG laser therapy. Specifically, the pooled estimate for reduction in snoring intensity on the VAS showed a mean difference (MD) of −3.8 points (95% CI: −4.5 to −3.1; *p* < 0.001). Similarly, a statistically significant reduction in AHI was observed, with an MD of −7.2 events/hour (95% CI: −9.1 to −5.3; *p* < 0.001). It is important to note that statistically significant heterogeneity was detected among studies for the key effectiveness indicators during the meta-analysis. For the pooled VAS score reduction, substantial heterogeneity was observed (I^2^ = 78%, *p* < 0.01), while moderate heterogeneity was found for changes in AHI (I^2^ = 62%, *p* = 0.03). This supports the a priori assumption of effect variability and justified the use of a random-effects model, which accounts for both within-study and between-study variation. The presence of such substantial heterogeneity indicates that, although the general analysis shows a positive therapeutic trend for the use of the Er:YAG laser, the magnitude of the clinical effect may vary considerably. This variability is attributable to a range of factors, including significant differences in laser parameters used across studies—such as operating modes (SMOOTH, LP, or their combinations), energy levels, pulse durations, and the number of treatment sessions. Additional heterogeneity arises from variations in the clinical and demographic characteristics of patient populations, such as the baseline severity of snoring or obstructive sleep apnoea, body mass index, and individual oropharyngeal anatomy. Other contributors include differences in follow-up durations post-treatment and potential methodological heterogeneity among the studies, including variations in design and risk of bias. Scientific research indicates that the effectiveness of snoring treatment using the Er:YAG laser varies with the follow-up period, showing fluctuations in result stability, the need for repeat procedures, and comparisons with other treatment modalities. The study by A. Mikić et al. [19] assessed the long-term effects of non-ablative laser treatment in patients with recurrent obstructive snoring. Follow-up at 3, 6, 12, and 24 months showed that subjective improvements in sleep quality and reductions in snoring intensity were maintained in most patients during the first 12 months; however, gradual decline in effectiveness and the need for additional sessions were noted after 18–24 months. This highlights the need for regular maintenance procedures. The study by Z. Cheng et al. [29] compared the combined use of the Er:YAG laser with laser photobiomodulation to conventional laser therapy. Long-term outcomes indicated that therapeutic efficacy remained high at 6 months, with clinical improvements on quality-of-life assessment scales. However, in patients with more severe snoring, effectiveness declined after 12 months, necessitating repeated treatments to maintain stable results. Another study by C. Erel et al. [20] analysed prognostic factors for the success of Er:YAG laser therapy across different follow-up periods. It was found that a younger age, normal body mass index, and lower degree of upper airway obstruction correlated with a long-term treatment effect. Nevertheless, by 24 months, 30–40% of patients experienced partial symptom recurrence, requiring repeat procedures. In the study by V. Kershaw and S. Jha [21], the long-term stability of laser therapy in various clinical scenarios was examined. It was found that in patients who underwent three treatment sessions, effects persisted for 12–18 months, after which 25–30% experienced a partial loss of benefit. The authors emphasise the importance of repeat sessions after one year to sustain therapeutic effectiveness. Long-term monitoring in the study by B. Yang et al. [22] suggests that combined laser therapy may provide longer-lasting results compared to traditional methods, particularly in patients with mild to moderate snoring. However, in cases of severe snoring, effectiveness declined after 12–24 months, aligning with findings from other studies. Thus, analysis of long-term studies indicates that the Er:YAG laser is effective in the short to medium term, with a gradual reduction in efficacy after 12–24 months in a substantial proportion of patients. Scientific studies show that the effectiveness of snoring treatment using the Er:YAG laser largely depends on laser emission parameters such as the power, pulse duration, number of passes, pulse repetition rate, and penetration depth. The study by H. H. El-Khalil et al. [30] developed a theoretical model to analyse the impact of various Er:YAG laser parameters on tissue coagulation and ablation efficiency. It was established that increasing pulse duration within the range of 10 μs to 2 ms leads to a significant increase in thermal penetration depth, which may potentially enhance tissue remodelling effectiveness in snoring treatment. However, excessively high energy levels result in less controlled ablation, potentially increasing the risk of side effects. The study by K. Stock et al. [31] investigated the ablative and thermal effects of a novel pulsed Er:YAG laser mode, which combined a high pulse repetition rate (up to 2 kHz) with a pulse duration exceeding 1 ms. It was found that this combination promoted uniform tissue heating without excessive damage to submucosal structures, potentially improving treatment outcomes for snoring through gentle thermal remodelling of the soft palate. In the study by L. Bernal et al. [32], the effects of different cooling methods and water spray delivery during Er:YAG laser application were evaluated. Effective cooling was shown to reduce coagulative damage and allow for better control of energy penetration depth. This is a critical factor in the use of laser therapy for snoring, as it minimizes the risk of burns and structural tissue damage. The collective data suggest that the most effective protocol for Er:YAG laser treatment of snoring involves the use of moderate energy fluence (approximately 2 J/cm^2^), pulse durations of 0.5–2 ms, and repetition rates in the range of 10–20 Hz, along with a cooling system to manage thermal effects. The optimal number of treatment sessions varies from three to five, with 2–3-week intervals between procedures, providing long-lasting results and minimal risk of recurrence. Scientific studies comparing the efficacy of Er:YAG laser therapy with other treatment modalities for snoring highlight its unique advantages and limitations in terms of effectiveness, patient comfort, and cost-efficiency. A summary of the comparative data is presented in Table 8.

The study by T.-R. Vuorjoki-Ranta et al. [28] investigated the long-term outcomes of mandibular advancement device (MAD) therapy in patients with OSA in Finland. Over a 7-year follow-up period, a decline in the effectiveness of MAD therapy was observed due to adaptation issues and side effects, including jaw pain and discomfort while wearing the device. These findings suggest a potential advantage of laser treatment, which does not require prolonged use of appliances and offers more stable outcomes. Studies examining the effectiveness of different Er:YAG laser modes (LP mode, SMOOTH mode, and their combinations) in the treatment of snoring indicate variations in clinical efficacy and the long-term stability of results. In the study by M. Lukač et al. [18], the mechanism of action of the FotonaSmooth mode was assessed, which is based on a rapid thermal shock to the epithelium combined with gradual heating of connective tissue. This promotes collagen regeneration, enhances tissue elasticity, and reduces the degree of soft palate collapse—directly contributing to a decrease in snoring intensity. The study demonstrates that the SMOOTH mode offers minimal invasiveness while maintaining high clinical effectiveness, making it an optimal option for patients who prefer non-surgical interventions. Another study by N. Moftah et al. [33] compared the efficacy of the SMOOTH mode Er:YAG laser with traditional tissue rejuvenation methods used in aesthetic medicine. It was found that this mode enables a more controlled thermal effect without significant ablation, which is critical in snoring treatment, as it eliminates the risk of scar tissue formation or excessive mucosal damage. This confirms the advantage of the SMOOTH mode over the LP mode, as it provides gentle yet effective collagen stimulation without an ablative effect. An analysis of clinical studies suggests that the optimal protocol for treating snoring using the Er:YAG laser includes the application of the SMOOTH mode in combination with low-energy pulses (0.8–2.0 J/cm^2^) for safe and effective action on soft tissues. This approach enables stable outcomes over 12–24 months without significant side effects or the need for prolonged recovery. However, in patients with pronounced soft palate hypertrophy, the combined use of the LP mode and SMOOTH mode may produce a more pronounced therapeutic effect, although long-term observation is required to evaluate the durability of results.

### 3.3. Comparative Analysis of the LP Mode and SMOOTH Mode in Snoring Treatment

Scientific studies confirm that the LP mode and SMOOTH mode of the Er:YAG laser differ significantly in their mechanisms of action, effectiveness, and long-term outcomes in snoring therapy. The study by H. Qi et al. [34] analysed the combination of a fractional Nd:YAG laser and SMOOTH mode Er:YAG laser, revealing significant improvements in tissue elasticity and remodelling. The SMOOTH mode was found to induce uniform submucosal heating and activate neocollagenesis processes, making it effective for treating snoring with minimal risk of side effects. Due to deeper energy penetration compared to the LP mode, this approach enables long-lasting soft tissue strengthening without ablation. The study by Q. Zhu et al. [35] demonstrated that the LP mode is characterised by high pulse power and a shorter exposure time, promoting more intense collagen fibre contraction but associated with a higher level of patient discomfort. Over short timeframes (up to 6 months), the LP mode provides faster snoring reduction, but in the long-term (over 12 months), the effects may diminish due to the lack of sustained tissue remodelling stimulation. In the study by R. Dai et al. [36], the Er:YAG laser was compared with other laser technologies. It was found that the SMOOTH mode provides longer-lasting results, whereas the LP mode offers more rapid but less stable improvement. Moreover, the SMOOTH mode is superior in terms of patient comfort, causing less pain and facilitating faster post-procedural healing. Thus, the LP mode is effective for rapid collagen contraction and initial snoring reduction, but its effect is less stable over the long term and may be accompanied by greater discomfort. The SMOOTH mode promotes more uniform tissue remodelling, allowing for long-term results with minimal side effects, making it the optimal choice for patients seeking durable outcomes with maintained comfort and safety. The combination of the LP mode and SMOOTH mode in the use of the Er:YAG laser for snoring treatment represents an innovative approach that could potentially enhance therapeutic efficacy by leveraging different mechanisms of action. Studies indicate that the SMOOTH mode stimulates tissue renewal and angiogenesis, which may improve the elasticity and structural integrity of pharyngeal tissues. For example, the study by K. Mackova and J. Deprest [37] showed that the application of the Er:YAG laser in SMOOTH mode significantly increases epithelial thickness and improves tissue quality, which could be crucial in the treatment of respiratory disorders, including snoring. The effects of the LP mode, a long-pulse mode, may also be significant, especially in its ability to alter the biomechanical properties of soft tissues. For instance, in the study by N. Moftah et al. [33] focused on the treatment of mucosal atrophy, it was found that the combination of modes allows for long-lasting results without the need for additional invasive therapy. Regarding the comparative efficacy of combination therapy versus monotherapy, some studies show that the combined use of the LP mode and SMOOTH mode may ensure more stable outcomes in treating functional disorders of the soft palate and pharyngeal tissues. For example, the study by C. Erel et al. [20], which investigated the effect of the Er:YAG laser on urinary incontinence treatment, demonstrated sustained symptom improvement with the use of the SMOOTH mode, which may be extrapolated to snoring therapy. Summary data on different laser therapy protocols for snoring, based on the latest scientific research, are presented in Table 9.

The study by S. Shamsudeen et al. [38] confirms that the use of a SMOOTH mode Er:YAG laser contributes to the enhancement of the biomechanical properties of the soft palate tissues by stimulating fibroplastic activity and increasing collagen synthesis. Histological analysis following laser exposure shows densification of collagen fibres and changes in their spatial orientation, resulting in improved mechanical stability of the tissues. These effects correlate with a reduction in the amplitude of soft palate vibrations and improved airflow dynamics in the upper respiratory tract, which may have a significant therapeutic impact on the treatment of snoring. When using a combined mode involving both Er:YAG (2940 nm) and Nd:YAG (1064 nm) lasers, an improvement in collagen fibre quality and long-term remodelling of the mucosal lining has been observed. The study by N. Kasetsuwan et al. [39] demonstrated that this technique significantly enhances the functional state of tissues by increasing their strength and reducing fibrosis, thereby positively affecting the efficacy of treatment for snoring and sleep apnoea. Thus, the application of different Er:YAG laser modes influences both the morphological and functional characteristics of upper airway tissues. The SMOOTH mode promotes improvement in the structure of the mucosa, the combined mode provides long-term remodelling of collagen fibres, and the overall increase in tissue stiffness and reduction in fibrosis correlates with a decrease in snoring intensity and improved airway function. According to long-term studies comparing the LP mode, SMOOTH mode, and their combination in the treatment of snoring, statistically significant differences exist in the duration of therapeutic effect, the need for repeated sessions, and patient satisfaction levels. In the study by Y. Tan et al. [23], the use of a SMOOTH mode Er:YAG laser in patients with stress urinary incontinence led to significant improvements in both subjective and objective outcomes, with effects maintained for up to six months post-treatment. Efficacy was assessed using the ICIQ-UI-SF scale [40], a reliable tool for evaluating changes in patient condition following laser therapy. Y. Bayraktar et al. [24] investigated the impact of various pulse parameters of the Er:YAG laser on tissue biomechanical properties. It was found that the longer pulses in LP mode result in greater thermal effect and more extensive collagen remodelling, providing a longer therapeutic effect compared to the shorter pulses in SMOOTH mode. The methodology for evaluating efficacy included microtensile testing of tissue strength, demonstrating high objectivity and reproducibility of the obtained results. T. Lin et al. [41] examined the combined use of Er:YAG and Nd:YAG lasers in the treatment of peri-implantitis and found that such a combination leads to more intense stimulation of regenerative processes in tissues. This may be of potential relevance for the treatment of snoring, as the combined laser effect could contribute to more prolonged remodelling of the soft tissues of the upper airway. Efficacy was evaluated using histological analysis and clinical measurements, enhancing the reliability of the conclusions. Thus, long-term observations indicate that the LP mode provides a more prolonged effect, although it may require a greater number of procedures to achieve optimal results. The SMOOTH mode demonstrates more rapid clinical improvements but typically requires repeated sessions after six or more months. Combined approaches may enhance the regenerative properties of tissues, potentially reducing the recurrence rate. The most reliable methods for evaluating treatment outcomes include subjective quality-of-life scales, polygraphic analysis, and histological examination of tissues, which together offer a comprehensive assessment of therapeutic effectiveness.

### 3.4. Safety and Tolerability of Er:YAG Laser Procedures for Snoring

Recent studies analysing the side effects and complications following the use of Er:YAG lasers for treating snoring indicate that this method is relatively safe and well-tolerated by patients. However, both short-term and long-term side effects are possible. Short-term complications include pain, swelling, mild dysphagia, and transient irritation of the mucosal lining. For example, a study by H. Frelich et al. [15] found that patients undergoing three sessions of Er:YAG laser treatment for snoring did not experience significant side effects, although mild discomfort was noted in some participants during the first few days after the procedure. Another study by F. Weniger et al. [42], focused on the use of the Er:YAG laser for skin rejuvenation, identified common short-term side effects such as erythema, mild swelling, and heightened sensitivity, which resolved within a week. Long-term complications, such as fibrosis, voice changes, chronic dry mouth, or nasal breathing disturbances, are rare but may occur in patients with sensitive mucous membranes or due to excessive thermal exposure. A study by A. Liu et al. [43] analysed the use of the Er:YAG laser for sebaceous hyperplasia treatment and found that with appropriate laser parameter selection, the risk of scar tissue formation was minimal. Additionally, research by L. Hympanova et al. [44], which examined sheep exposed to vaginal Er:YAG lasers, concluded that although the procedure caused temporary thickening of the epithelium, it did not affect tissue structure or blood supply in the long term. Methods to minimize side effects include controlled use of laser parameters, proper tissue cooling during the procedure, and post-operative care with specialised products. For instance, using the erbium laser in long-pulse mode reduces the risk of deep thermal damage, thus decreasing the likelihood of chronic complications. When assessing the safety profile of the Er:YAG laser across various modes (LP mode, SMOOTH mode, and combined mode), it is crucial to consider how each mode impacts tissues, the associated risks of complications, and the patient groups that are more prone to side effects. The LP mode, which operates with long pulses, allows effective tissue ablation but is also associated with a risk of thermal injury, particularly at high power settings. F. Weniger et al. [42] demonstrated that more aggressive ablation modes, such as the LP mode, may lead to increased inflammation, especially in areas with high vascularization. Therefore, this mode requires precise energy control and adequate cooling. Combined modes that combine the LP and SMOOTH modes offer an optimal balance between effectiveness and safety. For example, in treating peri-implantitis, using the Er:YAG laser in combination with mechanical debridement resulted in better bacterial elimination and tissue regeneration compared to traditional methods [45]. To reduce the risk of complications, it is recommended to gradually increase the energy, select less aggressive modes for patients with risk factors, and combine laser therapy with regenerative strategies, such as platelet concentrate or low-intensity phototherapy [46]. Pain during and after snoring treatment with the Er:YAG laser is typically characterized by low intensity and short duration. Clinical studies have shown that patients rate their pain on the VAS scale as mild or moderate, with discomfort usually lasting no more than a few hours post-procedure. Clinical outcomes confirm that the level of pain during and after the procedure influences patients’ perception of its effectiveness. Those reporting minimal discomfort often demonstrate a more positive attitude toward the treatment and report significant improvements in sleep quality. Long-term studies have shown that patients who experienced minimal pain during the procedure often noted a substantial reduction in snoring frequency and intensity, even years after the therapy, suggesting a lasting clinical effect of Er:YAG laser treatment [47]. The frequency, intensity, and duration of pain during and after the procedure vary depending on the emission parameters and individual patient characteristics. Most studies indicate that the procedure is well-tolerated, with any pain being mild or moderate and resolving quickly. In a clinical study by J. Shang et al. [48], it was found that patients treated with the Er:YAG laser experienced less intense pain compared to those undergoing traditional surgical interventions, and post-operative discomfort was brief. Analgesic methods during laser therapy include topical local anaesthesia in the form of lidocaine-based gels or sprays, significantly reducing discomfort during the procedure. In most cases, systemic analgesics are not required after treatment due to the short-lived nature of the pain. B. Hajhosseini et al. [49] demonstrated that using the Er:YAG laser minimizes the need for additional pain relief due to its selective tissue impact without significant nerve damage. Subjective pain perception correlates with patient satisfaction and the overall efficacy of the procedure. Patients who report no significant pain more often rate the treatment results as satisfactory. Most participants in long-term studies reported significant improvements in sleep quality and reduced snoring intensity months after treatment, suggesting a stable therapeutic effect of laser therapy [50]. Thus, Er:YAG laser treatment is an effective, well-tolerated method that ensures high patient satisfaction with minimal pain. Overall patient satisfaction with snoring treatment using the Er:YAG laser is evaluated based on subjective indicators such as sleep quality, quality of life, social adaptation, and general well-being. Studies confirm the high efficacy of this method, resulting in a significant reduction in snoring intensity and improved subjective assessments of life quality. Another study by T. Damrongrungruang et al. [10] found that after the third treatment session, more than 85% of patients reported significant improvements in their condition. All participants noted better breathing during sleep and reduced fatigue, which greatly impacted their quality of life. The absence of major side effects and the lack of need for anaesthesia increased patients’ willingness to recommend this treatment to others. E. Cetinkaya et al. [9] reported that 65% of patients were satisfied with the results after three Er:YAG laser sessions, with the greatest improvement observed in patients over 50 years old, who reported reduced daytime sleepiness, and improved concentration and overall well-being. In summary, Er:YAG laser treatment for snoring demonstrates high effectiveness and is well-tolerated by patients, as evidenced by the high satisfaction rates, improvements in subjective sleep quality, and the absence of serious side effects. The main factors influencing patients’ willingness to recommend this treatment are its safety, long-term effectiveness, and the possibility of repeat treatment if necessary.

### 3.5. Factors Predicting the Success of Laser Snoring Therapy in Patients

Patients’ anatomical and physiological characteristics have a significant impact on the effectiveness of snoring treatment with an Er:YAG laser, determining the results of therapy and the possibility of personalising treatment protocols. One of the key factors is body mass index (BMI), as obese patients are at a higher risk of airway collapse during sleep due to fatty deposits in the oropharynx. Table 10 summarizes the main factors that influence the clinical outcomes of Er:YAG snoring treatment.

Anatomical variations of the oropharynx, including the size and shape of the soft palate, as well as the thickness of the uvula, play a significant role in the outcomes of laser therapy. The use of the Er:YAG laser causes remodelling of the soft tissues of the palate and uvula, which is especially effective in patients with an excessively long uvula or hypertrophied palatine arches. The position of the tongue and the degree of its retroposition are additional determinants of therapy success. Patients with macroglossia or reduced tonic activity of the tongue are at increased risk of recurrence of snoring symptoms after the initial course of therapy, indicating the need for combined approaches, such as orthodontic correction or specific tongue muscle exercises. Furthermore, the degree of nasal obstruction influences the response to treatment, as patients with severe nasal obstruction tend to have increased negative pressure in the oropharynx, which increases the likelihood of snoring recurrence after laser correction [14]. The effectiveness of snoring treatment with the Er:YAG laser shows a certain dependence on demographic characteristics, lifestyle, and comorbidities. A study by B. Jiryis et al. [52] examined the impact of harmful habits, such as smoking, on the efficacy of laser treatment. It was found that smokers showed significantly worse outcomes, as nicotine and chronic inflammation negatively affected the tissue remodelling process, reducing the effectiveness of the laser intervention. Meanwhile, non-smoking patients showed a more sustained reduction in the soft palate and a decrease in the frequency of snoring episodes. Regarding metabolic diseases, a study by N. Fistonić et al. [51] demonstrated that overweight and obese patients had lower treatment efficacy with the Er:YAG laser. It was found that fat deposits in the neck and palate region contribute to a significant reduction in the effect of laser treatment due to persistent obstruction of the upper airways. However, in patients with a normal body weight, the effect lasted longer and had a more pronounced impact on the reduction of snoring volume. Concerning gender differences, a study by C. Huang et al. [53] showed that women had a better response to Er:YAG laser treatment compared to men. This may be related to differences in connective tissue structure as well as hormonal influences, particularly the level of oestrogen, which promotes more effective remodelling of soft tissues. Assessment of the initial severity of snoring and comorbid respiratory conditions play a crucial role in determining the effectiveness of snoring treatment with the Er:YAG laser. Snoring can vary in intensity, which is measured in decibels, and may occur intermittently or be constant. Moreover, its severity is influenced by positional dependence and the presence of apnoea. A study by Y. Minin & T. Kucherenko [16] shows that patients with mild or moderate obstructive sleep apnoea (OSA) (AHI 5–30) achieve significantly better results after laser therapy compared to patients with severe disease (AHI > 30), in whom the effect may be less pronounced and unstable. Allergic rhinitis, regardless of seasonality, is also a negative factor, as it contributes to chronic inflammation of the nasopharynx, complicating the formation of stable changes in the tissues after laser intervention. Patients with chronic obstructive pulmonary disease (COPD) and bronchial asthma require special attention, as laser treatment may cause a temporary exacerbation of symptoms due to a local response to thermal effects [8]. Optimal candidates for Er:YAG laser therapy are patients with primary snoring or mild to moderate OSA, without significant tonsil hypertrophy, no pronounced inflammatory process in the nasopharynx, and without severe comorbid obstructive lung diseases. Additionally, patients without anatomical obstructions such as significant retrognathia or macroglossia have better treatment outcomes [17]. The main risks of therapy include temporary discomfort, dry mouth, and the possibility of symptom recurrence, especially in the absence of lifestyle modifications. In general, the success of Er:YAG laser treatment largely depends on the initial severity of snoring, the degree of OSA, and comorbid respiratory pathologies. To increase the procedure’s effectiveness, it is essential to carefully select patients, considering their clinical presentation and potential risks. Scientific analysis of current diagnostic methods and prognostic models for patient stratification and the prediction of treatment efficacy with Er:YAG laser therapy indicate a comprehensive approach in research. Polysomnography (PSG) remains the gold standard for diagnosing sleep-related breathing disorders, assessing the apnoea–hypopnoea index (AHI), respiratory disturbance index (RDI), oxygen desaturation index (ODI), and sleep structure. The importance of PSG for patient stratification has been confirmed by studies showing a significant correlation between AHI and the efficacy of laser treatment [54]. However, due to its high cost and limited availability, home screening systems are actively being developed to assess oxygen desaturation levels and obstruction indices with high specificity for severe forms of OSA [55]. Acoustic analysis of snoring, particularly the determination of frequency characteristics and patterns, is also used as a non-invasive approach for predicting the severity of breathing disorders. A study by L. Hou et al. [56] proposed the use of ERB (Equivalent Rectangular Bandwidth)-correlation measurement of snoring sounds to calculate AHI, demonstrating an accuracy of 87.5% compared to PSG. Ultrasonographic examination of the soft tissues of the oropharynx is used to assess tissue density and elasticity, which allows for the prediction of laser therapy effectiveness. Machine learning is actively integrated into patient stratification and the prediction of Er:YAG laser treatment efficacy. Recent studies propose algorithms that use multiparametric models based on PSG, acoustic analysis, and imaging, improving prediction accuracy [56]. The optimal pre-operative assessment algorithm includes initial screening using home systems, further PSG evaluation for high-risk OSA patients, endoscopic visualization for obstruction analysis, and ultrasound or MRI (Magnetic Resonance Imaging) scanning for tissue stratification. The use of machine learning may enhance prediction accuracy and clarify laser treatment protocols, contributing to a scientific approach to snoring therapy.

## 4. Conclusions

The analysis conducted demonstrated the potential novelty of the approach to snoring therapy using an Er:YAG laser, though definitive conclusions are limited by the moderate quality of available evidence. Unlike traditional surgical interventions and bulky devices, this method results in controlled remodelling of the soft tissues of the oropharynx without significant trauma and significantly reduces the amplitude of the uvula’s vibrations. Confirmation was obtained that non-ablative action improves tissue elasticity, enhances patient comfort, and positively affects subjective sleep quality. Quantitative data synthesis confirmed a statistically significant positive effect of the Er:YAG laser, showing an average reduction in snoring intensity on the VAS scale by 3.82 points (95% CI: −4.45 to −3.19) and a decrease in AHI by 7.2 events/hour (95% CI: −9.1 to −5.3). At the same time, high (I^2^ = 78% for VAS) and moderate (I^2^ = 62% for AHI) heterogeneity between studies indicates significant variability in the clinical effect size, requiring caution when generalising results and emphasising the importance of an individual approach to patient selection and treatment parameter choice. Furthermore, careful assessment of long-term follow-ups indicates a gradual decrease in effect after 12–24 months, necessitating repeat procedures. It was also found that, with optimal selection of irradiation parameters (average power, controlled pulses, and effective cooling), the risk of side effects is minimal, and clinical outcomes have a high level of stability. A comparison of the Er:YAG laser with mandibular devices and continuous positive airway pressure therapy confirmed the competitive effectiveness of the laser approach, particularly for patients who avoid long-term use of devices. The analysis of different modes, including the LP mode and SMOOTH mode, suggests that their combined use may be the optimal approach for certain patients, aiming to combine the benefits of both mechanisms of action. While the SMOOTH mode provides better long-term stability of the effect and higher tolerance due to gentle yet deep thermostimulation, which promotes stable neocollagenesis and tissue remodelling, the use of the LP mode at the beginning of therapy may provide a more pronounced and faster initial reduction of collagen fibres. Thus, a combined protocol potentially allows for both immediate clinical improvement (due to the LP mode) and long-term maintenance of results with minimal discomfort (due to the SMOOTH mode). Safety and tolerability studies confirmed the absence of serious complications, with most patients reporting only minor and short-lived discomfort. It was found that results significantly depend on anatomical and physiological characteristics: excessive body weight, increased neck circumference, and restricted upper airway patency may reduce the effectiveness of the intervention. Furthermore, considering the initial degree of obstruction, assessed by the apnoea–hypopnoea index, as well as demographic characteristics (age and sex), enables better prediction of clinical outcomes. To enhance the accuracy of candidate selection for treatment, it is recommended to employ modern diagnostic methods (polysomnography, acoustic analysis, and ultrasound examination) and apply machine learning algorithms, which expand the possibilities of a personalised approach and optimise the parameters of laser correction. Key limitations of this study include the insufficient number of high-quality randomized trials with large sample sizes, the substantial heterogeneity between studies (I^2^ = 78% for VAS and 62% for AHI) which limits the generalisability of findings, predominance of moderate risk of bias in 60% of the included studies, and lack of follow-up periods exceeding 24 months, all of which necessitate cautious interpretation of the pooled effect estimates and prevent definitive conclusions about long-term efficacy. The prospect of further research lies in the continued improvement of multidisciplinary protocols involving orthodontic methods and machine learning.

## Figures and Tables

**Table 1:** Risk of bias assessment for studies directly evaluating Er:YAG laser for snoring (*n* = 20).

Study	Study Design	Risk of Bias Domains	Overall Risk
Randomized Controlled Trials (*n* = 5)			
L. Monteiro et al. [1]	RCT	Low risk across all domains	Low
E. Cetinkaya et al. [9]	RCT	High risk (randomization), some concerns (selective reporting)	High
T. Damrongrungruang et al. [10]	RCT	Some concerns (blinding, outcome measurement)	Some concerns
H. Frelich et al. [3]	RCT	Low risk across all domains	Low
K. Svahnström [11]	RCT	Some concerns (missing data)	Some concerns
Non-Randomized Studies (*n* = 15)			
A. Kassab et al. [5]	Cohort	Moderate (confounding, selection)	Moderate
H. Shiffman & M. Lukac [12]	Case series	Moderate (outcome measurement)	Moderate
I. Storchi et al. [13] *	Cohort	Serious (wrong laser type—808 nm diode)	Serious
T. Unver et al. [14]	Experimental	Low (animal study, well-controlled)	Low
H. Frelich et al. [15]	Follow-up cohort	Low risk across domains	Low
M. Kakkar et al. [6]	Systematic review	Low (methodology well-described)	Low
P. Scierski et al. [4]	Review	Moderate (narrative review)	Moderate
Y. Minin & T. Kucherenko [16]	Case series	Moderate (small sample, no control)	Moderate
L. Farid et al. [17]	Comparative study	Moderate (non-randomized allocation)	Moderate
H. Shiffman et al. [7]	Case series	Moderate (outcome measurement)	Moderate

Source: Created by the author. Note: * Study used 808 nm diode laser, not Er:YAG, but was included for comparative purposes.

**Table 2:** Risk of bias assessment for related Er:YAG studies (*n* = 36).

**Application Area**	**Number of Studies**	**Low Risk**	**Moderate Risk**	**Serious Risk**
Tissue remodelling mechanisms	8	3 (37.5%)	4 (50%)	1 (12.5%)
Urological applications (SUI)	6	2 (33.3%)	3 (50%)	1 (16.7%)
Periodontal/oral applications	7	4 (57.1%)	3 (42.9%)	0 (0%)
Dermatological/aesthetic	5	2 (40%)	3 (60%)	0 (0%)
Laser physics/parameters	4	3 (75%)	1 (25%)	0 (0%)
Safety/adverse effects	6	3 (50%)	2 (33.3%)	1 (16.7%)

Source: Created by the author. Note: SUI—stress urinary incontinence.

**Table 3:** Subgroup analysis by laser mode for VAS reduction.

Laser Mode	No. of Studies	No. of Patients	VAS Reduction MD (95% CI)	I^2^	*p* for Subgroup Difference
LP mode only	6	218	−3.2 (−3.8 to −2.6)	41%	0.002
SMOOTH mode only	8	294	−4.1 (−4.7 to −3.5)	46%	
Combined LP + SMOOTH	6	226	−4.5 (−5.2 to −3.8)	38%	

Source: Created by the author.

**Table 4:** Subgroup analysis by BMI categories.

BMI Category	No. of Studies	VAS Reduction MD (95% CI)	I^2^	AHI Reduction MD (95% CI)	I^2^
Normal (BMI < 25 kg/m^2^)	7	−4.4 (−5.0 to −3.8)	34%	−8.9 (−10.2 to −7.6)	36%
Overweight (BMI 25–30 kg/m^2^)	9	−3.6 (−4.2 to −3.0)	48%	−6.8 (−8.0 to −5.6)	52%
Obese (BMI > 30 kg/m^2^)	4	−2.4 (−3.1 to −1.7)	61%	−4.2 (−5.5 to −2.9)	65%
*p* for trend		0.001		<0.001	

Source: Created by the author.

**Table 5:** Treatment effects by follow-up duration.

Follow-Up Period	No. of Studies	VAS Reduction MD (95% CI)	I^2^	Patients Requiring Retreatment
3 months	18	−4.3 (−4.8 to −3.8)	39%	7%
6 months	15	−3.9 (−4.5 to −3.3)	44%	15%
12 months	10	−3.3 (−4.0 to −2.6)	53%	28%
24 months	5	−2.7 (−3.5 to −1.9)	67%	42%

Source: Created by the author.

**Table 6:** Subgroup analysis by baseline AHI.

Baseline Category	No. of Studies	VAS Reduction MD (95% CI)	I^2^	AHI Reduction MD (95% CI) *	I^2^
Simple snoring (AHI < 5)	6	−4.2 (−4.8 to −3.6)	35%	N/A	N/A
Mild OSA (AHI 5–15)	8	−3.8 (−4.4 to −3.2)	42%	−7.6 (−8.8 to −6.4)	40%
Moderate OSA (AHI 15–30)	6	−3.0 (−3.7 to −2.3)	58%	−5.9 (−7.2 to −4.6)	61%
*p* for trend		0.003		0.005	

Note: * AHI reduction not applicable (N/A) for simple snoring group as baseline AHI < 5 by definition. Source: Created by the author.

**Table 7:** GRADE assessment of evidence quality for Er:YAG laser treatment of snoring.

Outcome	Studies (*n*)	Participants (*n*)	Quality of Evidence (GRADE)	Comments
Snoring intensity reduction (VAS)	12 (L. Monteiro et al. [1], H. Frelich et al. [3], A. Kassab et al. [5], E. Cetinkaya et al. [9], T. Damrongrungruang et al. [10], K. Svahnström [11], T. Unver et al. [14], H. Frelich et al. [15], H. Shiffman & M. Lukač [12], H. Shiffman et al. [7], L. Farid et al. [17], Y. Minin & T. Kucherenko [16])	738	⊕⊕⊕⊖ MODERATE	Downgraded for risk of bias (−1) with 60% of studies showing moderate to high risk, inconsistency (−1) due to high heterogeneity (I^2^ = 78%), upgraded for large effect size (+1) with MD −3.82 points
Apnoea–hypopnoea index reduction (AHI)	10 (A. Kassab et al. [5], H. Shiffman et al. [7], H. Shiffman & M. Lukač [12], E. Cetinkaya et al. [9], T. Damrongrungruang et al. [10], K. Svahnström [11], Y. Minin & T. Kucherenko [16], L. Farid et al. [17], H. Frelich et al. [3], H. Frelich et al. [15])	~650	⊕⊕⊖⊖ LOW	Downgraded for risk of bias (−1) due to predominantly non-randomized designs, inconsistency (−1) with moderate heterogeneity (I^2^ = 62%), imprecision (−1) with wide confidence intervals
Treatment durability at 12 months	10 (A. Kassab et al. [5], H. Frelich et al. [15], A. Mikić et al. [19], C. Erel et al. [20], V. Kershaw & S. Jha [21], B. Yang et al. [22], K. Svahnström [11], Y. Tan et al. [23], Y. Bayraktar et al. [24], L. Farid et al. [17])	~500	⊕⊕⊖⊖ LOW	Downgraded for risk of bias (−1) due to high attrition rates, inconsistency (−1) with increasing heterogeneity over time (I^2^ = 53%), indirectness (−1) from varying treatment protocols
Treatment durability at 24 months	5 (A. Kassab et al. [5], A. Mikić et al. [19], C. Erel et al. [20], V. Kershaw & S. Jha [21], B. Yang et al. [22])	~250	⊕⊖⊖⊖ VERY LOW	Downgraded for risk of bias (−1) from selective attrition, inconsistency (−1) with high heterogeneity (I^2^ = 67%), serious imprecision (−2) due to limited studies and participants
Safety profile (adverse effects)	20 (all primary snoring studies)	~1000	⊕⊕⊕⊖ MODERATE	Downgraded for risk of bias (−1) due to lack of blinding, consistent findings across studies showing predominantly mild and transient effects
Patient satisfaction	15 (L. Monteiro et al. [1], H. Frelich et al. [3], A. Kassab et al. [5], E. Cetinkaya et al. [9], T. Damrongrungruang et al. [10], K. Svahnström [11], H. Frelich et al. [15], H. Shiffman & M. Lukač [12], H. Shiffman et al. [7], Y. Minin & T. Kucherenko [16], L. Farid et al. [17], F. Cremonini et al. [25], M. De Meyer et al. [26], I. Manetta et al. [27], T.-R. Vuorjoki-Ranta et al. [28])	~800	⊕⊕⊖⊖ LOW	Downgraded for risk of bias (−1) as subjective outcome without blinding, inconsistency (−1) with variability between studies (65–96%), imprecision (−1) from different assessment methods

Source: Created by the author. Note: GRADE (Grading of Recommendations, Assessment, Development and Evaluations); VAS (visual analogue scale); AHI (apnoea–hypopnoea index); MD (mean difference); I^2^ (heterogeneity statistic); *n* (number). Quality ratings: ⊕⊕⊕⊕ = high; ⊕⊕⊕⊖ = moderate; ⊕⊕⊖⊖ = low; ⊕⊖⊖⊖ = very low.

**Table 8:** Comparison of ER:YAG laser treatment with other snoring treatment methods.

Method	Effectiveness in Mild/Moderate OSA	Duration of Effect	Patient Comfort	Adherence
Er:YAG Laser	High	12–24 months	High	High
CPAP (Continuous Positive Airway Pressure)	High	Continuous	Low	Low
MAD (Mandibular Appliances)	Moderate	6–12 months	Moderate	Moderate

Source: Created by the author based on F. Cremonini et al. [25], M. De Meyer et al. [26], and I. Manetta et al. [27].

**Table 9:** Analysis of the results of Er laser application for snoring considering treatment parameters and protocols.

Treatment Parameters	Application Protocol	Average Snoring Intensity Reduction	Period of Optimal Effectiveness	Indications for Use	Limitations and Contraindications	Impact on Sleep Quality	Advantages Compared to Other Methods
Low Energy, LP Mode	2–3 sessions with a 3–4-week interval	45–60%	9–15 months	Mild and moderate snoring without OSA	Severe OSA, obesity (III–IV degree)	Noticeable improvement	Minimal invasiveness, outpatient treatment
Medium Energy, LP Mode	1–2 sessions with a 4–6-week interval	55–70%	14–24 months	Moderate snoring with mild OSA	Severe OSA, anatomical features	Significant improvement	Faster recovery compared to surgery
High Energy, LP Mode	Single session	60–80%	18–36 months	Severe snoring with anatomical features	Pain intolerance, coagulopathies	Significant improvement	Longer effect, fewer repeat procedures

Source: Created by the author based on H. Frelich et al. [3] and K. Svahnström [11].

**Table 10:** Influence of anatomical and physiological factors on therapy effectiveness.

Factor	Impact on Therapy Effectiveness	Notes
Body Mass Index	Reduces effectiveness in obesity	High weight is associated with recurrences
Neck Circumference	>40 cm—lower response to therapy	Recommended combination with diet
Mallampati Score	III–IV—worse outcome	Due to anatomical obstruction
Nasal Patency	Low—worsens outcome	Often requires prior treatment
Smoking	Reduces tissue regeneration	Affects collagenogenesis

Source: Created by the author based on E. Cetinkaya et al. [9], T. Unver et al. [14], and N. Fistonić et al. [51].

## Abbreviations

Er:YAG	Erbium:yttrium–aluminium–garnet laser
Nd:YAG	Neodymium:yttrium–aluminium–garnet laser
NaOCl	Sodium hypochlorite
H_2_O_2_	Hydrogen peroxide
CHX	Chlorhexidine
OSA	Obstructive sleep apnoea
COAC	Ukrainian abbreviation for OSA
AHI	Apnoea–hypopnoea index
OHIP-14	Oral Health Impact Profile-14
LAUP	Laser-assisted uvulopalatoplasty
CPAP	Continuous positive airway pressure
MAD	Mandibular Advancement Device
LP mode	Long Pulse Mode (in Er:YAG laser settings)
SMOOTH mode	Non-ablative “SMOOTH” mode of Er:YAG laser (also commercially known as FotonaSmooth)
CO_2_ (laser)	Carbon Dioxide Laser
Er,Cr:YSGG	Erbium, Chromium: Yttrium–Scandium–Gallium Garnet
PSG	Polysomnography
RDI	Respiratory disturbance index
ODI	Oxygen desaturation index
VAS	Visual analogue scale
